# Case report: Rethinking NGS analysis in diagnosing Diamond-Blackfan anemia syndrome

**DOI:** 10.3389/fgene.2024.1459291

**Published:** 2024-11-01

**Authors:** Panayiota L. Papasavva, Konstantinos Kaouranis, Stefania Byrou, Constantina G. Constantinou, Iacovou Efrosini, Marina Kleanthous, Carsten W. Lederer, Thessalia Papasavva

**Affiliations:** ^1^ Department of Molecular Genetics Thalassemia, The Cyprus Institute of Neurology & Genetics, Nicosia, Cyprus; ^2^ Platonas Medical Center, Apostolos Loukas Medical Center, Nicosia, Cyprus; ^3^ ECCLabs-Independent Cytopathology Services (IHCS), Nicosia, Cyprus

**Keywords:** diamond-blackfan anemia syndrome, whole exome sequencing, next generation sequencing, bone marrow failure syndrome, data analysis, filtering

## Abstract

Diamond-Blackfan anemia syndrome (DBAS) is a rare inherited bone marrow failure (BMF) syndrome characterized by erythroid aplasia, congenital malformations, and cancer predisposition. With its genetic heterogeneity, variable penetrance and expressivity, DBAS poses significant diagnostic challenges, necessitating advancements in genetic testing for improved accuracy. Here, we present the case of an 18-year-old male with a long-standing macrocytic anemia that remained undiagnosed despite standard whole exome sequencing (WES). Revisiting a family-trio WES analysis with clinical insight led to the identification of a likely pathogenic variant in the Ribosomal Protein S17 (*RPS17*) gene, previously masked due to analytical challenges and conservative filter settings. This variant, an initiation codon mutation, was confirmed in heterozygosity in both the proband and his mother through Sanger sequencing. Comprehensive imaging studies showed no malformations or organ anomalies in either individual, except for mild esophageal stenosis observed in both. *RPS17* mutations, particularly those affecting the initiation codon, have previously been linked to the DBAS phenotype, but strong pathogenic association has not yet been firmly established. Our case warns of potential underdiagnosis of *RPS17* variants in DBAS, highlighting the importance of clinical context and interdisciplinary collaboration in interpreting WES data to avoid false-negative results.

## 1 Introduction

Diamond-Blackfan anemia syndrome (DBAS) is a rare, inherited bone marrow failure (BMF) syndrome characterized by erythroid aplasia, congenital malformations and an increased risk of cancer. It follows an autosomal dominant inheritance pattern with variable expressivity and incomplete penetrance, but most cases are sporadic (Hattangadi and Lipton, 2018; Costa et al., 2020). DBAS exhibits significant genetic heterogeneity, with mutations in ribosomal protein-coding genes, particularly those causing haploinsufficiency of these proteins, being the most prevalent cause. About 25% of DBAS cases are linked to *RPS19* gene mutations (Idol et al., 2007), while other variations such as point mutations, deletions, and copy number variations affect genes encoding both large (*RPL5*, *RPL11*, *RPL35A*, *RPL26*, *RPL15*) and small (*RPS26*, *RPS10*, *RPS24*, *RPS17*, *RPS7*, *RPS28*, *RPS29*) ribosomal subunits (Ulirsch et al., 2018). In a minority of cases, other genes such as *GATA1*, *HSP70*, *TSR2* and *CECR1* are involved (Costa et al., 2020).

Recent advancements in genetic testing have begun to clarify the relationship between ribosomal protein haploinsufficiency, and DBAS pathogenesis. The “ribosomal stress” hypothesis suggests that decreased ribosomal protein synthesis activates p53, leading to cell cycle arrest or apoptosis and to DBAS features and symptoms (Costa et al., 2020; Liu and Ellis, 2006; Farrar et al., 2011).

The diagnostic criteria for DBAS have evolved to become more gene-centric, now also encompassing individuals who exhibit minimal or no symptoms of the disease if they carry DBAS-associated genetic variants (non-classical or “silent” cases) (Wlodarski et al., 2024; Vlachos and Muir, 2010; Steinberg-Shemer et al., 2016). This broadening facilitates improved surveillance, informed family planning and identification of suitable hematopoietic stem cell transplantation donors, particularly important given the variable disease expressivity, incomplete penetrance and increased cancer risk in these individuals (Vlachos et al., 2008). Modern techniques, such as whole exome sequencing (WES), whole genome sequencing (WGS), and combined DNA-RNA sequencing, have significantly improved the power of molecular diagnoses.

In this report, we detail the diagnostic challenges encountered in an 18-year-old male patient who presented with long-standing macrocytic anemia. He was born full-term to healthy non-consanguineous Caucasian parents (Figure 2A). Developmental milestones were attained normally, but his height consistently remained below the 5th percentile. During adolescence he was diagnosed with attention-deficit/hyperactivity disorder but received no medication. He did not exhibit susceptibility to infections or gastrointestinal symptoms, except for occasional oral aphthous ulcers. From infancy, he had a history of low hemoglobin (Hb) levels, and low white blood cell (WBC) counts, with no need for transfusions or pharmacological intervention.

**FIGURE 1:**
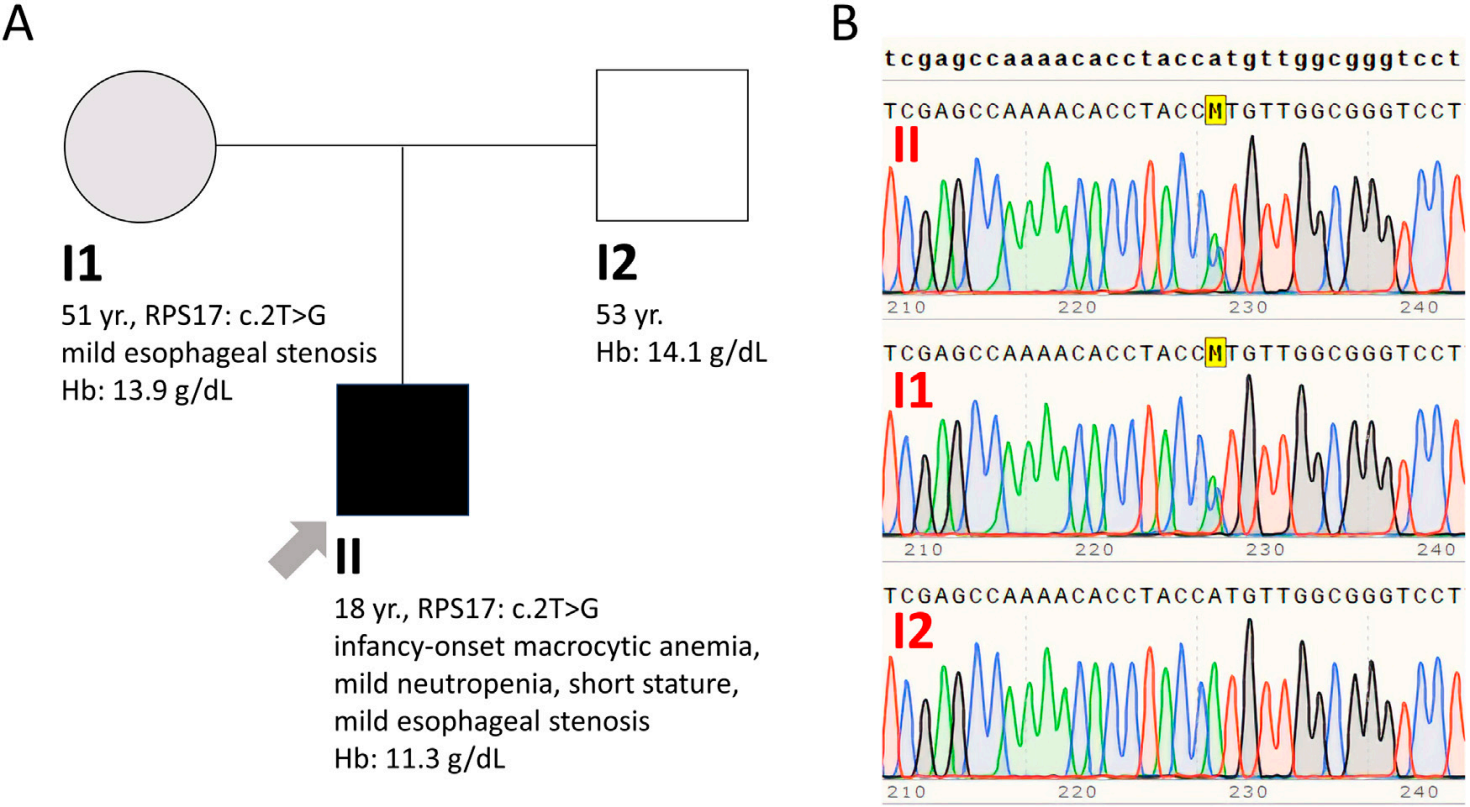
Bone marrow aspiration smear **(A)** and trephine biopsy **(B)** showing hypocellularity for age with decreased erythroid precursors, presence of megaloblasts, and normal, maturing myeloid and megakaryocytic cell lineages, with no dysplastic features.

On presentation, a short stature of 161 cm was noted, but the physical examination was otherwise unremarkable. Laboratory assessment revealed mild macrocytic anemia and mild neutropenia. The Hb level was 11.3 g/dL (normal range 14–18 g/dL), hematocrit was 34% (normal range 38%–52%), with a mean corpuscular volume of 110 fL (normal range 80–98 fL) and a mean corpuscular hemoglobin of 36.5 pg (normal range 26–32 pg). WBC count was 3,550/μL (normal range 4,500–11,000/μL), with 47% neutrophils, 40% lymphocytes, 11% monocytes and 2% eosinophils. The platelet count was normal, and the reticulocyte index was 0.9 (<2 indicating impaired red blood cell formation). Levels of glucose, renal, hepatic and thyroid function tests, as well as B12 and folate, haptoglobin, lactate dehydrogenase and bilirubin, all fell within normal limits. Coombs test was negative. Ferritin was 304 ng/mL (normal range 30–400 ng/mL), and transferrin saturation was 48% (normal range 15%–45%). A bone marrow examination was performed, revealing marked hypocellularity for age, with decreased erythroid precursors, presence of megaloblasts, and normal, maturing myeloid and megakaryocytic cell lines, with no dysplastic features (Figure 1). Bone marrow flow cytometry showed a distorted myeloid maturation pattern with 0.9% of total cells being myeloid progenitors and 1.7% early monocytoid cells. A next-generation-sequencing (NGS) tumour-based (somatic) panel evaluating single-nucleotide variants, indels, and copy number variations in 75 genes associated with myeloid and lymphoid malignancies yielded negative results. Chromosomal analysis showed a 46, XY karyotype with no evidence of any chromosomal abnormality. Erythrocyte adenosine deaminase (eADA) levels, elevated in 75%–80% of DBAS-affected individuals, were within normal limits, whereas erythropoietin levels were very high at 2,135.9 mIU/mL (normal range 2.6–18.5 mIU/mL). A bone marrow WES analysis performed in another centre did not reveal a causative variant.

Prompted by this diagnostic impasse, the patient was referred to our center for advanced study by an interdisciplinary team, including geneticists and a hematologist, and a new family-trio-based WES analysis was conducted from peripheral blood. After routine filtering and analysis steps did not yield significant findings, the strong clinical suspicion of a BMF-related disorder prompted us to revisit the raw sequencing data for BMF-associated variants via WES *in silico* analysis based on a customized analysis pipeline with relaxed filter criteria.

**FIGURE 2:**
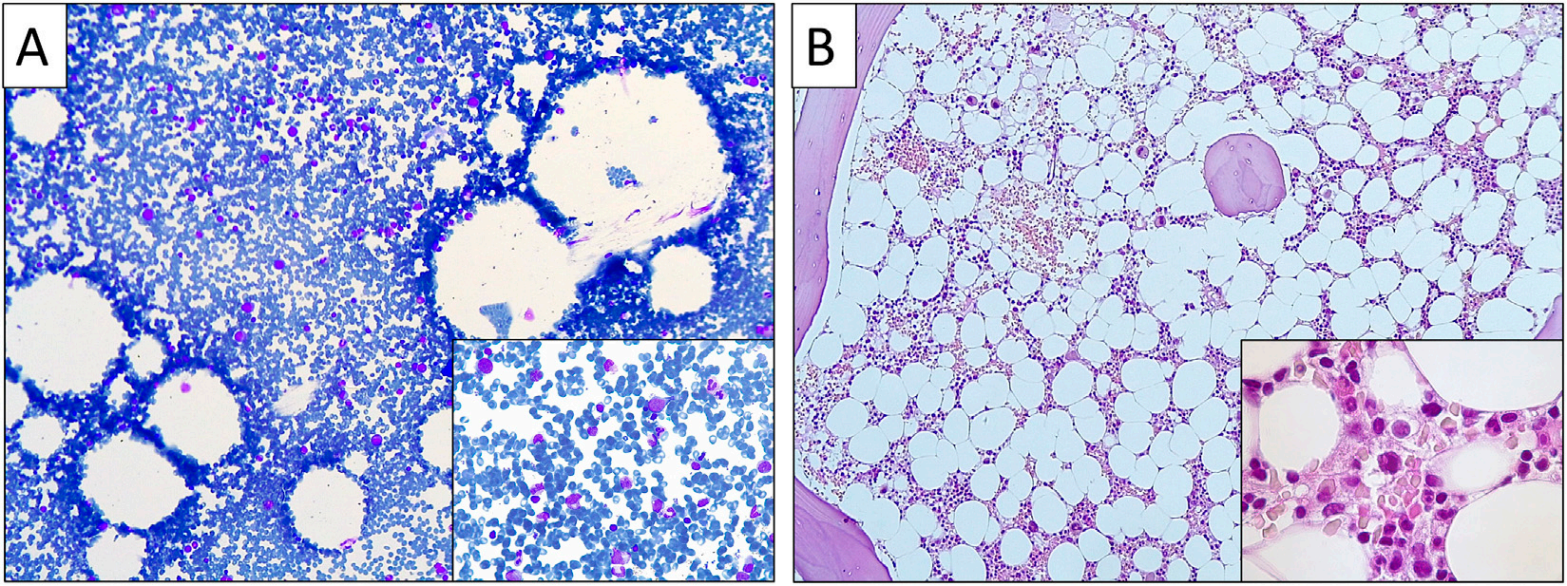
**(A)** Family pedigree. Clinically affected son is shown in black shape, mildly affected mother in grey shape, and clinically (and genetically) unaffected father in white shape. **(B)** Sanger sequencing analysis confirming the mother and son as heterozygous for the RPS17 (NM_001021.3): c.2T>G (p.Met1Arg) variant.

This approach resulted in the identification of the NM_001021.6 (RPS17):c.2T>G (p.Met1Arg) variant in the Ribosomal Protein S17 (*RPS17*) gene (rs116840811) in both the proband and mother (allelic balance 0.41 and 0.45, respectively), which had initially been filtered out due to low coverage (0.17 and 0.20, respectively) below our threshold of >0.20. This variant is classified in ClinVar as pathogenic (review status one gold star: criteria provided, single submitter), and in VarSome Clinical platform as likely pathogenic. The *RPS17* gene, located on chromosome 15, spans five exons over a length of 3.7 kb and codes for the RPS17 protein, a vital structural component of the small ribosomal subunit. In the context of translation initiation, small ribosomal subunit proteins, such as RPS19 and RPS24, are known to interact with the eukaryotic initiation factor eIF2, yet the specific role of RPS17 in normal erythrocyte development and the effects of *RPS17* mutations on erythropoiesis in DBAS are still not fully understood. The *RPS17* gene, typical for genes encoding ribosomal proteins, has multiple processed pseudogenes dispersed through the genome (NCB1, 2024), which often impedes the application of standard sequence mapping and in consequence leads to low-quality data for parts of the gene. This issue frequently leads to the exclusion of this gene from NGS panels (Blueprint Genetics, 2024). Previous studies have indicated that there are four functional copies of the *RPS17* gene and suggested the possibility of individual variability in the sensitivity to changes in *RPS17* copies (Farrar et al., 2011). To verify the mutation, Sanger sequencing was employed, confirming the proband and mother as heterozygous for the *RPS17* (NM_001021.3): c.2T>G (p.Met1Arg) variant (Figure 2B). This variant affects the translation initiation codon and is predicted to result in either loss of translation initiation or N-terminal truncation (pathogenic very strong 1 (PVS1) criterion according to the American College of Medical Genetics and Genomics (ACMG) and the Association for Molecular Pathology (AMP) published standards and guidelines for the interpretation of sequence variants) (Richards et al., 2008). Of note, the bone marrow exome analysis previously performed by another laboratory, which initially failed to identify the variant, was revisited, confirming the presence of the variant in both the proband and his mother. Combining our genetic findings with the clinical presentation and the available knowledge, a diagnosis of DBAS was strongly considered. Subsequently, comprehensive imaging studies were conducted for both mother and son to rule out congenital organ anomalies. Cardiac/urinary malformations were absent, however, gastroscopy detected mild esophageal stenosis in both, a common finding in BMF syndromes, but more prevalent in congenital dyskeratosis than in DBAS. Neither individual experienced dysphagia. The mother’s complete blood count and stature measurements were normal. Given the potential for cancer predisposition, iron overload and other complications associated with DBAS, ongoing careful surveillance will be implemented for both individuals to monitor their health and detect any emerging issues early.

## 2 Materials and methods

### 2.1 Sample collection and DNA extraction

Peripheral blood was collected from the proband and parents in EDTA-containing tubes. Genomic DNA was extracted using the MagCore^®^ Genomic DNA Whole Blood Kit (RBC Bioscience, Taipei, Taiwan) and quantified using Qubit fluorometer.

### 2.2 WES analysis

Library preparation was conducted following the Illumina DNA prep with Enrichment protocol (Illumina, San Diego, CA, United States), utilizing the Illumina Exome panel targeting protein-coding regions and exon-intron boundaries (±25 bp). Sequencing was performed on the NextSeq2000 desktop sequencer (Illumina, United States) using the P3 Reagent kit (Illumina, San Diego, CA, United States), generating paired-end 100 bp reads. Variant discovery, annotation, and interpretation were carried out using the Varsome Clinical platform (CE IVD) with the human reference genome build hg19. Initially, standard analysis filters were applied, filtering out variants with a population frequency of 0.01 and above, as well as variants with fewer than 20 sequencing reads aligned to the variant position and/or an allele balance lower than 0.22. These filters were removed for re-analysis of data.

### 2.3 Sanger sequencing

The RPS17 gene reference sequence was obtained from the NCBI database (accession number NC_000015.9, GRCh37.p13). Forward (CCT​AAG​CTT​TAA​CAG​GCT​TCG) and reverse (GGG​ACG​ATT​GTG​GAG​GAT​G) primers were designed using SnapGene software. Conventional PCR was performed as follows: 95°C for 5 min; 28 cycles at 95°C for 30 s, 58°C for 30 s, 72°C for 30 s; and 72°C for 7 min. PCR purification was performed using EXO-SAP (New England Biolabs, Ipswich, MA, United States), followed by cycle sequencing using the BigDye Terminator v1.1, cycle sequencing kit and protocol (Applied Biosystems, Foster City, CA, United States). Products were analyzed on an automated Genetic Analyzer (Applied Biosystems 3500XL, Foster City, CA, United States).

## 3 Discussion

Here we identified a family case suggestive of DBAS linked to the RPS17 (NM_001021.3): c.2T>G (p.Met1Arg) variant, following an initial failure to identify relevant genetic aberrations based on standard WES data processing and analyses. Due to the presence of 16 pseudogene copies as paralogs in the human genome, the candidate gene *RPS17* poses analytical challenges (low coverage or allele balance), leading to its deliberate exclusion or inadvertent oversight from diagnostic genetic panels.

Identifying diagnostically significant variants amid a vast array of non-significant ones in exome and genome datasets remains a major analytical challenge, requiring a considerable amount of effort and time. The use of custom filters to narrow the number of candidate variants and accurately identify those that are truly causal is, thus, a standard and routine stage of data analysis pipelines, commonly referred to as the “filtering/prioritization” step. Initial strategies include implementing quality control filters that address challenges in alignment and variant calling, eliminating low-quality reads and concentrating on high-confidence variants. Also, filters are applied on standard parameters such as sequencing depth, allele balance, and population allele frequency, with the latter specifically used to exclude common variants and focus on those linked to rare diseases (Pedersen et al., 2021). Additional strategies include assignments of pathogenicity and filtering by segregation, if multiple affected family members are sequenced, or by inheritance patterns. However, these filtering and prioritization techniques carry the risk of inadvertently eliminating variants that are potentially associated with the phenotype, as initially occurred in the present case (Gilissen et al., 2012). Improved bioinformatic pipelines and access to genomic variant databases with accurate and thoroughly curated data enhance sensitivity and specificity of analyses. Yet, interpretation of genomic data should not be solely dependent on technological prowess and advancements, but also on detailed clinical data and interdisciplinary collaboration between geneticists, clinicians and researchers (Baptista, 2022). For many unsolved cases, revisiting raw data, alignments and coverage reports with additional contextual information may lead to diagnoses. In our case, clinical insights guided a targeted approach, prompting the removal of filters that masked phenotypically matched candidate genes and leading to an important finding. The provision of detailed clinical information to laboratories by referring physicians is crucial for accurate diagnosis, especially for known causative genes, such as *RPS17* in DBAS cases. In such instances, initial tentative diagnoses via WES can then be validated by more targeted techniques, such as Sanger sequencing, for reliable diagnoses.

Interestingly, *RPS17* (NM_001021.3): c.2T>G (p.Met1Arg) was historically the first mutation linked to DBAS in the *RPS17* gene, discovered in 2007 in a patient with severe macrocytic anemia, facial dysmorphy and short stature. For that patient, the mutation was determined to be *de novo* and was shown to cause haploinsufficiency in RPS17 production, with basal translation at 39–47% of controls (Cmejla et al., 2007). Based on the PVS1, PS2, PS4, PP4, BS4, BP4 criteria of the ACMG/AMP published standards and guidelines for the interpretation of sequence variants, the variant was then classified as pathogenic (Durkie et al., 2024). However, more advanced functional studies are needed to establish a causal impact on protein function (PS3). On another occasion, a different *de novo* mutation in the *RPS17* initiation codon (c.1A>G) was identified in a Korean infant with severe macrocytic anemia, red cell aplasia, and growth retardation (Song et al., 2010). Both patients required treatment with chronic blood transfusions as well as iron chelation. In our family case, the proband exhibits a mild phenotype, while the mother shows almost no phenotype, which can be explained by previous indications of individual variability in sensitivity to *RPS17* dose (Farrar et al., 2011). Moreover, DBAS and specifically mutations in ribosomal proteins have been shown to exhibit variable expressivity and incomplete penetrance, and it is possible that other genetic factors might contribute to the proband’s milder phenotype compared to previously reported cases, as well as the almost unaffected phenotype observed in the mother. Reports for mutations in *RPS17* are extremely rare, with a total of seven pathogenic variants reported in the 2023-12-03 version of ClinVar, two of which are copy number variations, specifically, large deletions (ClinVar, 2024). However, our case indicates that the challenge in obtaining high-quality sequencing data for this gene, and its occasional omission from related panels, may lead to underdiagnosis of *RPS17* mutations, suggesting that their prevalence among DBAS patients could be significantly higher than currently recognized based on standard analysis protocols. This underscores the importance of specifically considering this gene and being mindful of its potential inadvertent removal from the analysis output in diagnostic evaluations for DBAS.

## Data Availability

Full data availability would compromise anonymity of the rare index case. Please contact the corresponding authors to request access to the dataset based on a corresponding confidentiality agreement.
